# Gonadotropin-releasing hormone (GnRH)-I and GnRH-II induce cell growth inhibition in human endometrial cancer cells: Involvement of integrin beta3 and focal adhesion kinase

**DOI:** 10.1186/1477-7827-7-81

**Published:** 2009-08-05

**Authors:** Dong Wook Park, Kyung-Chul Choi, Colin D MacCalman, Peter CK Leung

**Affiliations:** 1 Department of Obstetrics and Gynecology, Child and Family Research Institute, The University of British Columbia, Vancouver, British Columbia, V6H 3V5, Canada; 2 Laboratory of Veterinary Biochemistry and Immunology, College of Veterinary Medicine, Chungbuk National University, Cheongju, Chungbuk, 361-763, Republic of Korea; 3 Laboratory of Reproductive Biology and Infertility, Cheil General Hospital and Women's Health Center. College of Medicine, Kwandong University, 100-380, Seoul, Republic of Korea

## Abstract

Endometrial carcinoma is the most common neoplasm of the female genital tract, accounting for nearly one half of all gynecologic cancers in the Western world. Although intensive research on pathological phenomena of endometrial cancer is currently going on, but exact cause and biological aspects of this disease are not well described yet. In addition to well-documented roles of gonadotropin-releasing hormone (GnRH) in hypopituitary ovarian (HPO) axis, the agonistic or antagonistic analogs (or both) of GnRH have been shown to inhibit the proliferation of a variety of human gynecologic cancers. Thus, in the present study, we further examined the possibility that GnRH induces integrin beta3 and activation of focal adhesion kinase (FAK) through mitogen-activated protein kinases (MAPKs), ERK1/2 and p38, to inhibit the growth of HEC1A endometrial cancer cell line. As a result, both GnRH-I and GnRH-II resulted in a significant increase in integrin beta3 expression and evoked the activation of FAK in a time-dependent manner in these cells. In addition, these analogs induced an activation of ERK1/2 and p38 MAPK in a time-dependent manner as downstream pathways of FAK. It appears that GnRH-II has much greater effect on the activation of FAK, ERK1/2 and p38 compared to GnRH-I in these cells. Further, we demonstrated that the growth inhibition of HEC1A cells by GnRH-I or GnRH-II is involved in the activation of integrin-FAK and ERK1/2 and p38 MAPK pathways. Taken together, these results suggest that GnRH may be involved in the inhibition of endometrial cancer cell growth via activation of integrin beta3 and FAK as a direct effect. This knowledge could contribute to a better understanding of the mechanisms implicated in the therapeutic action of GnRH and its biomedical application for the treatment against endometrial cancer.

## Background

Endometrial carcinoma is the most common neoplasm of the female genital tract, accounting for nearly one half of all gynecologic cancers in the Western world. It is estimated that approximately 40,000 new cases of endometrial cancer are diagnosed annually in the United States and about 7,000 women die of this disease, indicating that endometrial carcinoma is thus the fourth most common malignancy and the eighth leading cause of cancer-related death in women [1]. Although intensive research on pathological phenomena of endometrial cancer is currently going on, but exact cause and biological aspects of this disease are not well elucidated yet.

Gonadotropin-releasing hormone (GnRH) is the hypothalamic hormone that mediates reproductive competence [2,3]. An intermittent GnRH secretion from the hypothalamus acts upon its receptor in the anterior pituitary to regulate the production and release of two gonadotropins, luteinizing hormone (LH) and follicle-stimulating hormone (FSH). In addition to reproductive roles of GnRH in hypo-pituitary ovarian (HPO) axis, GnRH-I, a classical form of GnRH, has an inhibitory effect on cell growth in human mammary, ovarian, endometrial, and prostate tumors and has been implicated as an antiproliferative factor of gynecologic cancers [4-8]. In particular, the agonistic or antagonistic analogs (or both) of GnRHs have been shown to inhibit the proliferation of a variety of human ovarian cancer cell lines in a dose- and time-dependent manner through activation of extracellular-signal regulated kinase (ERK) and p38 [9,10]. A second form of GnRH, GnRH-II, was expressed at the transcriptional level and GnRH-II induced an inhibition of the ovarian cancer cell growth in our previous study [11]. In addition, several in vitro investigations showed that GnRH agonists and the GnRH antagonist, i.e., cetrorelix, can inhibit the proliferation of Ishikawa and HEC1A human endometrial cancer lines and primary cultures of human endometrial cancer [12-15].

Integrins modulate intracellular signals by growth factor receptors and play a central role in cell migration. An integrin heterodimer generally consists of noncovalently linked α- and β-subunits, each subunit having a large extracellular domain, a single membrane spanning domain and a short, noncatalytic cytoplasmic tail. There are 18 α and β subunits that form at least 25 distinct pairs of α and β heterodimers with different ligand specificity [16,17]. In addition to the previously reported antiproliferative effect, integrin α plays an important role in the migratory/invasive behavior of melanoma cells expressing GnRH receptors [18]. In addition to controlling cell adhesion and shape, integrins also transmit signals either by physical association with several growth factor receptors or directly through recruitment of non-receptor tyrosine kinases from the focal adhesion kinase (FAK) and Src families [19]. Although molecular events of integrins have been recently uncovered in various cell types, their role in tumorigenesis is yet to be defined. Thus, it is of interest to examine whether or not integrins and FAK may be involved in GnRH-induced growth inhibition in endometrial cancer cells. In the present study, we further investigated effect of GnRH on the proliferation of HEC1A endometrial cancer cell line, through integrin and its downstream effecter molecules, i.e., FAK and mitogen-activated protein kinases (MAPKs).

## Methods

### Reagents

A GnRH-I analog, Trp (6), was purchased from Sigma-Aldrich Ltd. (St Louis, MO, USA). A GnRH-II analog, d-Arg(6)-Azagly(10)-NH2, was purchased from Peninsula Laboratories (Belmont, CA). A p38 inhibitor, SB202190, was purchased from Sigma-Aldrich Ltd.

### Antibodies

The polyclonal anti-integrin β3, polyclonal anti-phospho-FAK (Ser-722), and monoclonal anti-FAK antibodies were purchased from Santa Cruz Biotechnology, Inc. (Santa Cruz, CA). The monoclonal anti-phospho-p44/42 MAPK (Thr202/Tyr204) and polyclonal anti-p44/42 MAPK antibodies were obtained from Cell Signaling Technology (Beverly, MA).

### Cell culture

A well-characterized human endometrial adenocarcinoma cell line, HEC1A, was purchased from the American Type Culture Collection (ATCC, HTB-112; Manassas, VA). These carcinoma cells were maintained according to the provider's instruction. The cells were then adjusted to DMEM/F12 (without phenol red, Invitrogen, Ontario, Canada) with 10% fetal bovine serum (FBS; Hyclone, USA). The culture medium was changed every 3 days and the cells were subdivided every 7 or 8 days. All treatments were performed after 4 h serum starvation and maintain serum free condition for appropriate experimental durations to remove effect of undefined endogenous hormones or cytokines. The cells were cultured on 1: 16 diluted (vol/vol) growth factor-reduced Matrigel (BD, Franklin Lakes, NJ) coated cell culture dish. The cells were treated with a specific inhibitor of p38, SB202190 (100 nM), for 20 min after removal of GnRH-1 and -2 containing medium.

### Immunoblot analysis

The cells treated with GnRH-I or -II were washed once with ice-cold PBS and lysed in 100 μl of in ice-cold RIPA buffer [150 mm NaCl, 1% Nonidet P-40, 0.5% deoxycholate, 0.1% sodium dodecyl sulfate (SDS), 50 mm Tris (pH 7.5), 1 mm phenylmethylsulfonyl fluoride, 10 μg/ml leupeptin, and 100 μg/ml aprotinin]. The extracts were placed on ice for 10 min, collected into a 1.5 ml tube, and centrifuged for 10 min at 19,000 G. The supernatants were moved to new tubes, and the concentration of supernatants was determined using Bradford assay (Bio-Rad Laboratories, Mississauga, Ontario, Canada). Thirty-five μg of total protein was mixed with 1/6 volume of sample buffer (75 mm Tri-HCl of pH 6.8, 15% SDS, 0.15% bromophenol blue, 15% glycerol, and 37.2% 2-mercapthoethanol) and boiled for 10 min. The sample mixture was run on 10% SDS-PAGE gels (acrylamide : bisacrylamide, 29:1) in gel running buffer (25 mm Tris/250 mm glycine, pH 8.3/0.1% SDS) at 100 V for 2.5 h and electrotransferred to a nitrocellulose membrane (Hybond C, Amersham Pharmacia Biotech Inc., Oakville, Ontario, Canada) at 100 V for 1.5 h. The membrane was immunoblotted using primary antibodies for overnight. After washing three times with TBS-T (0.1% Tween 20 in Tris-buffered saline) for 15 min, the signals were detected with horseradish peroxidase-conjugated secondary antibody (Amersham Pharmacia Biotech Inc.), and visualized using the enhanced chemiluminescence system (Amersham Pharmacia Biotech Inc.). The intensity of signals was quantitated by densitometry (Bio-DocAnalyze, Biometra, Germany).

### Proliferation assay

Proliferation assay was performed using [^3^H]thymidine incorporation assay as previously described [20]. Briefly, 20,000 cells were seeded in 24-well plates and cultured in 0.5 ml medium. GnRH-I and -II were diluted appropriately with medium, and the cells were cultured 24 h. The medium was changed after 24 h incubation. After treatment, the cells were then incubated with medium containing 1 μCi [^3^H]thymidine (0.5 Ci/mmol; Amersham Pharmacia Biotech Inc.) and collected after 6 h incubation. The cells were washed three times with PBS and precipitated with 0.5 ml 10% trichloroacetic acid for 20 min at 4°C. The precipitate was washed in methanol twice and solubilized in 0.5 ml of 0.1 N sodium hydroxide. The radioactivity was measured in the Tri-Carb Liquid Scintillation Analyzer (Model 2100TR, Packard Instrument Co., Meriden, CT).

### Statistical analysis

Data were subjected to ANOVA test. Each experiment was repeated three times in duplicate or triplicate. Data are shown as means of three individual experiments and presented as the mean ± S.D. Expression level of proteins are shown as fold changes compared with control levels. [^3^H]Thymidine incorporation assay was presented as the percentage of growth compared with control level and as the mean ± S.D. 10 measurements were performed per experiment for each condition. *P *< 0.05 was considered statistically significant.

## Results

### Antiproliferative effect of GnRHs

**Figure 1:**
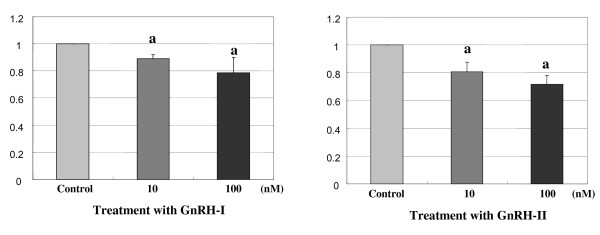
**Effect of GnRH-I and GnRH-II on the cell growth**. HEC1A cells were treated with GnRH-I or GnRH-II analog (10 nM or 100 nM) for 24 h in serum-free medium. Cell proliferation was determined by ^3^H-thymidin incorporation assay following treatment with GnRHs. Values are the means of cell number (± S.E.) in triplicates of three independent experiments. a, indicates *p *< 0.05 vs. control (cont).

To determine the effect of GnRH-I and -II on the cell proliferation, the HEC1A cells were treated with increasing doses of GnRH-I or -II (10 and 100 nM) for 24 h. As seen in Fig. 1**(left panel)**, treatment with GnRH-I resulted in a significant decrease in cell proliferation compared to control. After treatment with GnRH-I for 24 h, the inhibitory effect of proliferation was observed at these concentrations of GnRH-I; 89.8 ± 1.1% at 10 nM and 79.2 ± 2.1% at 100 nM, respectively. In addition, GnRH-II also induced cell growth inhibition of HEC1A cells at the doses of GnRH-II; 80.2 ± 1.8% (10 nM) and 71.2 ± 2.2% (100 nM), respectively (Fig. 1; **right panel**). These results indicate that HEC1A cell proliferation is dose-dependently decreased by GnRH-I and -II at these concentrations.

### Effect of GnRHs on the expression of integrin β3 subunit

**Figure 2:**
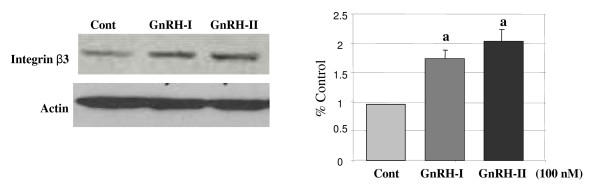
**Effect of GnRH-I and GnRH-II on the expression of integrin β3 subunit**. HEC1A cells were treated with GnRH-I or GnRH-II analog (100 nM) for 24 h in serum-free medium. The protein level of integrin β3 subunit was determined and measured by immunoblot analysis. Data are shown as means of three individual experiments and presented as the mean ± S.D. a, indicates *p *< 0.05 *vs*. control (cont).

To determine the effect of GnRH-I and -II on the expression of integrin β3, the HEC1A cells were treated with GnRH-I or -II (100 nM) for 24 h and then the protein level of integrin β3 was measured by immunoblot analysis. As demonstrated in Fig. 2, treatment with GnRH-I resulted in a significant increase in integrin β3 expression compared to control. In similar, treatment with GnRH-II induced a marked increase in the protein level of integrin β3 in these cells (Fig. 2). These results indicate that the effect of both GnRH-I and GnRH-II on the inhibition of cell proliferation may be involved in the expression of integrin β3 protein in endometrial cancer cells.

### Activation of FAK by GnRH-I and -II

**Figure 3:**
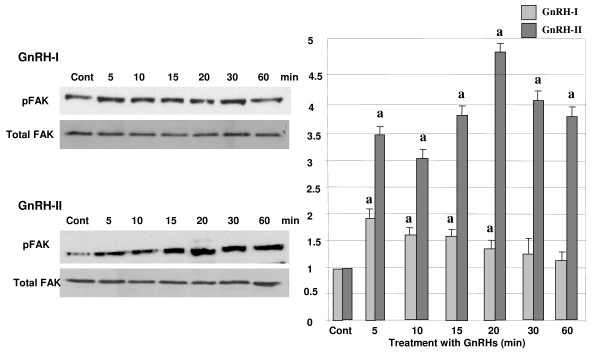
**Effect of GnRH-I and GnRH-II on the activation of FAK**. HEC1A cells were treated with GnRH-I or GnRH-II (100 nM) in a time-dependent manner. Phosphorylation of FAK was determined by Western blot analysis following treatment with GnRHs. Data are shown as means of three individual experiments and presented as the mean ± S.D. a, indicates *p *< 0.05 *vs*. control (cont). The white bars indicate GnRH-I treatment while gray bars indicate GnRH-II treatment.

To investigate whether GnRH-I and -II induce the activation of FAK *in vitro *as a downstream pathway of integrin signaling, the cells were treated with GnRH-I or -II (100 nM) in a time-dependant manner (0, 5, 10, 15, 20, 30, and 60 min) following 4 h serum starvation. Following treatments with GnRH-I or GnRH-II, phosphorylated form of FAK (pFAK) was measured using a specific antibody to phosphorylated FAK by immunoblot analysis. In addition, total form of FAK was measured by this method and pFAK was normalized by the expression of total FAK. As shown in Fig. 3, the treatment with GnRH-I resulted in a significant increase in the phosphorylation of FAK in HEC1A cells. It was of interest that GnRH-II induced much greater expression of pFAK in these cells compared to GnRH-I as seen in Fig. 3, indicating that GnRH-II may have a greater effect on the activation of pFAK compared to that of GnRH-I in HEC1A endometrial cancer cells.

### Activation of ERK1/2 by GnRH-I and -II

**Figure 4:**
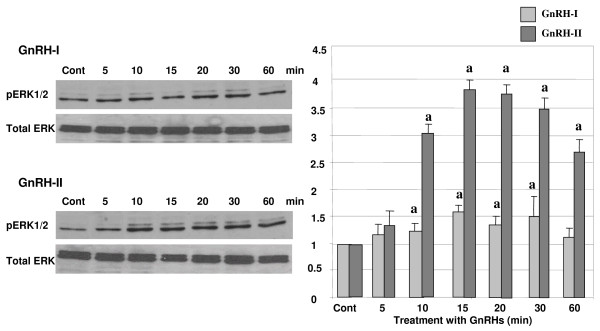
**Effect of GnRH-I and GnRH-II on the activation of ERK1/2**. HEC1A cells were treated with GnRH-I or GnRH-II (100 nM) in a time-dependent manner. Phosphorylation of ERK1/2 was determined by Western blot analysis following treatment with GnRHs. The *error bars *represent the mean ± S.D. from three independent experiments. a, indicates *p *< 0.05 *vs*. control (cont).). The white bars indicate GnRH-I treatment while gray bars indicate GnRH-II treatment.

To investigate whether GnRH-I and -II induce the activation of ERK1/2, the cells were treated with GnRH-I and -II (100 nM) in a time-dependant manner (0, 5, 10, 15, 20, 30, and 60 min) after 4 h serum starvation. A phosphorylated form of ERK1/2 (pERK1/2) was measured using a specific antibody to phosphorylated ERK1/2 by immunoblot analysis. In addition, total form of ERK1/2 was measured and pERK1/2 was normalized by the expression of total ERK1/2. The treatment with GnRH-I resulted in a significant increase in the phosphorylation of ERK1/2 in HEC1A cells (Fig. 4). In addition, GnRH-II induced much greater expression of pERK1/2 in these cells compared to GnRH-I as seen in Fig. 4, indicating that GnRH-II may have a greater effect on the activation of pERK1/2 compared to that of GnRH-I in HEC1A cells.

### Activation of p38 by GnRH-I and -II

**Figure 5:**
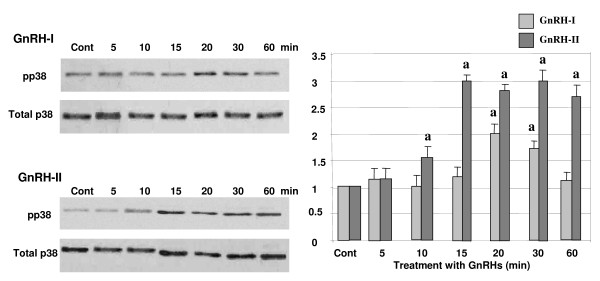
**Effect of GnRH-I and GnRH-II on the activation of p38**. HEC1A cells were treated with GnRH-I or GnRH-II (100 nM) in a time-dependent manner. Phosphorylation of ERK1/2 was determined by Western blot analysis following treatment with GnRHs. The *error bars *represent the mean ± S.D. from three independent experiments. a, indicates *p *< 0.05 *vs*. control (cont).). The white bars indicate GnRH-I treatment while gray bars indicate GnRH-II treatment.

**Figure 6:**
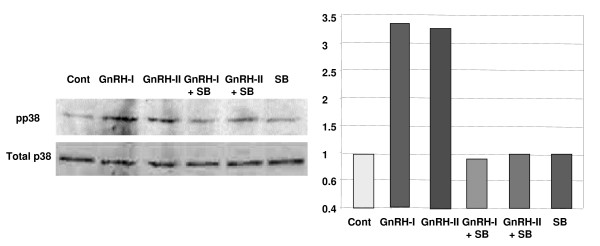
**Effect of SB202190 on the GnRH-I-induced or GnRH-II-induced p38 activation**. HEC1A cells were treated with GnRH-I or GnRH-II for 20 min in serum-free medium in the absence or presence of SB202190 (100 nM), a specific inhibitor of p38, to further elucidate the direct effect of GnRH-I or -II on the activation of p38 in HEC1A cells.

To investigate whether GnRH-I and -II induce activation of p38, the cells were treated with GnRH-I and -II (100 nM) in a time-dependant manner (0, 5, 10, 15, 20, 30, and 60 min) after 4 h serum starvation. As shown in Fig. 5, GnRH-I significantly phosphorylated p38 MAPK (pp38) following treatment for 20 min, while GnRH-II showed much stronger and rapid effect on the activation of p38 than GnRH-I after 10 to 60 min. To further elucidate the direct effect of GnRH-I or -II on the activation of p38 in HEC1A cells, the cells were pretreated with a p38 inhibitor, SB202190 (100 nM), followed by treatment with GnRH-I or -II (100 nM) for 20 min. As seen in Fig. 6, pretreatment with SB202190 reversed GnRH-I or -II induced phosphorylation of p38, whereas no significant difference was observed in the cells treated with only SB202190 itself.

### Involvement of p38 pathway in GnRH-I and -II-induced cell growth

**Figure 7:**
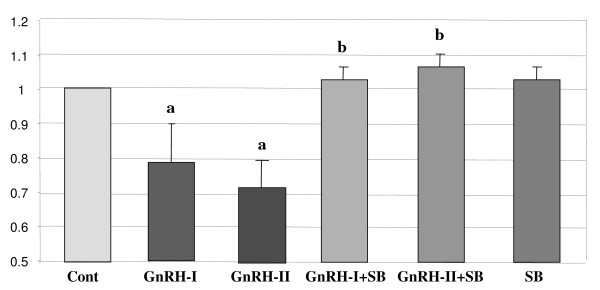
**Effect of SB202190 on the GnRH-I- or GnRH-II-induced inhibition of cell growth**. HEC1A cells were treated with GnRH-I or GnRH-II for 24 h in serum-free medium in the absence or presence of SB202190 (100 nM), a specific inhibitor of p38. Cell proliferation was determined by ^3^H-thymidin incorporation assay following treatment with GnRHs. Values are the mean cell number (± S.E.) from three independent experiments. Values are the means of cell number (± S.E.) in triplicates of three independent experiments. a, indicates *p *< 0.05 vs. control (cont); b, indicates *p *< 0.05 *vs*. GnRH-I or GnRH-II treatment only.

To further investigate the involvement of p38 MAPK pathway in the cell growth inhibition by GnRHs, HEC1A cells were treated with GnRH-I analog (100 nM) or GnRH-II analog (100 nM) for 24 h in serum-free medium in the absence or presence of SB202190 (100 nM), a specific inhibitor of p38, and cell proliferation was determined by ^3^H-thymidin incorporation assay. The treatment with GnRH-I or GnRH-II resulted in a marked decrease in cell growth in these cells, while pretreatment with SB202190 completely abolished the antiproliferative effect of GnRH-1 or -II as demonstrated in Fig. 7.

## Discussion

In addition to the hypothalamus, GnRH-I has also been localized to the endometrium, placenta, breast, ovary, testis and prostate [2,21-23]. In the endometrium and myometrium, GnRH receptor has been detected by a radioligand-binding assay and immunohistochemistry [21]. Furthermore, Engel et al., (2005) showed that the cytotoxic GnRH analogues AN-152 and AN-207 inhibit growth of xenografted HEC1A and RL-95-2 human endometrial carcinoma cell lines into nude mice [1]. In the present study, GnRH-I and -II inhibit the cell growth of HEC1A human endometrial cancer cell line. In addition, treatment with increasing doses of GnRH-I and -II (100 nM) resulted in the much stronger inhibition than 10 nM treatment. Thus it would be assumed that HEC1A cell proliferation is dose-dependently decreased by GnRH-I and -II at these concentrations.

A further support for GnRH influence on cell proliferation was provided in a previous study [24]. Davidson et al. demonstrated that activation of the GnRH receptor resulted in both cell adhesion and cytoskeletal remodeling and GnRH (10^-7 ^M) increased adhesiveness of HEK293 cells overexpressing GnRH receptor. A cytoskeletal remodeling was dependent on FAK, c-Src, ERK and Rac and independent from the classic phospholipase C signaling pathway [24]. GnRH-dependent intracellular signaling events for downstream of PKC have been characterized [25]. Several groups have demonstrated that GnRH receptor occupancy resulted in the activation of ERK and c-*jun *N-terminal protein kinase [26,27], members of the MAPK superfamily. GnRH activation of the JNK cascade is dependent on the low molecular weight GTP-binding protein, Cdc42 [26]. In addition to the ERK and JNK cascades, the MAPK superfamily includes the p38 kinase pathway [20,27,28] reported that GnRH can stimulate activation of the p38 kinase pathway. Activation of the p38 kinase by GnRH requires PKC, suggesting that GnRH-induced p38 MAPK activation may selectively contribute to the regulation of c-*fos *protooncogene expression, but not c-*jun *or the glycoprotein hormone α-subunit gene. Recently, we demonstrated that binding of GnRH-I (a classical form of GnRH) and GnRH-II (a second form of GnRH) to the GnRH-I receptor activates ERK1/2 through a PKC-dependent pathway and is essential for GnRH-induced anti-proliferation of ovarian cancer cells [20]. In this study, we clearly demonstrated that GnRH-I or GnRH-II resulted in a significant increase in the phosphorylation of ERK1/2 and p38 in HEC1A cells, suggesting these MAPKs are involved in GnRH-I or GnRH-II induced cell growth inhibition of endometrial cancer cells. It is of interest that GnRH-II induced much greater expression of pERK1/2 or pp38 in these cells compared to GnRH-I, suggesting that GnRH-II may have a greater effect on the activation of pERK1/2 or pp38 compared to that of GnRH-I in HEC1A endometrial cancer cells.

Immunohistochemical studies for human endometrium throughout the menstrual cycle revealed a sudden increase in β3-integrin expression in luminal and glandular epithelial cells at mid secretory phase [29,30]. It was of interest that the overexpression of integrin β3 subunit suppressed tumor growth [31]. So far, ligand binding and clustering are known as an important step for full integrin function and the recruitment of downstream signal transduction cascade molecules [32]. Numerous studies have suggested that β3 and αVβ3 integrins are overexpressed in solid tumors, and that this overexpression plays a role in tumor growth and invasion. It was speculated that β3 overexpression in glioma cells suppresses, rather than stimulates, glioma growth, but that this occurs exclusively *in vivo *and not *in vitro *[31]. Previous studies revealed that the growth and metastasis of transplanted tumors in mice showed lacking of specific cell adhesion receptors, integrins or selectins [20,33,34]. In addition, integrin β3 or selectin null mice showed increased primary tumor growth *in vivo *[35]. In this study, GnRH-I and -II can increase integrin β3 expression in HEC1A cells *in vitro*, suggesting that GnRH may be a direct regulator of integrin β3 expression and play a pivotal role as a growth inhibitor by regulating its expression in endometrial cancer cells.

It is also apparent that the GnRH receptor, like many other heptahelical G protein-coupled receptors, can also activate monomeric G protein molecules [36]. The activation of these monomeric pathways has been shown to mediate important effects of GnRH in gonadotropes such as control of gonadotropin hormone subunit transcription [26]. A previous study has also shown that in many cell types, stimulation of Gi- or Gq-coupled receptors causes FAK activation [37-39]. FAK also has been reported to bind to the intracellular regions of β-integrin subunits, and to play a pivotal role as a signal integrator downstream of cell-ECM interactions and other receptor and non-receptor tyrosine kinases [40]. In this study, we demonstrated that GnRH induced FAK phosphorylation in HEC1A endometrial cancer cells. GnRH-I induced FAK phosphorylation at 5 min and decreased within 30 min. On the other hand, GnRH-II increased FAK phosphorylation after 5 min and reached maximum rate at 20 min following treatment. Based on these observations, it can be suggested that activation of FAK in HEC1A cells by GnRH is mediated by elevated integrin β3 expression. FAK has known to be a regulator of ERK activation in many cell types [24,41]. Phosphorylation of ERK was induced by treatment with GnRH-I and -II in this study as shown in the Results. Many substrates of ERK are localized in the nucleus and cytosol. In addition, ERK phosphorylates many cytoskeletal elements and is important for the determination of cellular morphology, and can serve as anchor proteins that direct ERK to their proper locations. In this study, we did not see any changes of cellular morphology and adhesion property in these cells (Data not shown). Thus, we suggest that ERK might interact with nuclear substrates such as transcriptional factors rather than cytoplasmic substrates such as cytoskeletal elements.

In our previous study, we demonstrated that the antiproliferative effect of GnRH-II in ovarian cancer cells may involve p38 MAPK, which led us to investigate the role of other MAPK family members [9]. To investigate the signal pathway involved in the antiproliferative effect by GnRH-I and -II, the activation of p38 in HEC1A cells was examined by immunoblot analysis using the phospho-specific MAPK antibody after treatment with GnRH-I and -II. The p38 MAPK is activated by the phosphorylation on tyrosine 180 and tyrosine 182 in the activation loop and modulates cell cycle for the response to environmental stress, hormones, ligands that bind to G protein-coupled receptors, and inflammatory cytokines [42]. In this study, we observed that GnRH-II (100 nM) induced the activation of p38 MAPK in as early as 10 min, which is in agreement with a previous study performed in OVCAR-3 cells [20], while GnRH-I showed weaker and delayed effect than GnRH-II. Furthermore, the activation of p38 MAPK was completely blocked by SB203580 (100 nM), a specific inhibitor of p38 MAPK, in this study. In addition to this, antiproliferative effect of GnRH was reversed by pretreatment of SB203580 (100 nM). Activation of p38 MAPK can also induce apoptotic cell death, but we did not detect any evidence of apoptosis in HEC1A cells following treatment with GnRH (Data not shown). This result was supported by previous data using HEC1A cells, indicating that GnRH analogs did not affect cell viability after treatment [43]. It can be speculated that effects of Antide, a GnRH receptor antagonist, may have some agonistic or antagonistic effect on the cell proliferation of endometrial cancer cells. Thus, Antide was treated with GnRH or alone for same duration abovementioned in HEC1A cells. In this study, we failed to observe any agonistic or antagonistic effect of Antide on the proliferation of HEC1A cells (data not shown). It was reported that Antide showed an agonistic effect on HEC1A cell proliferation only after 6-day treatment, while no significant antiproliferative effect was observed at earlier times [43]. This result indicates that Antide may have a lower affinity to GnRH receptors than GnRH analogs. So far, an anti-tumor effect of GnRH analogs has been reported. We speculated that both GnRH-I and -II analogs resulted in an inhibition of human endometrial cancer cell proliferation and this effect is mediated by GnRH receptor and its related signal transduction molecules.

In summary, both GnRH-I and GnRH-II resulted in a significant increase in integrin β3 expression and evoked the activation of FAK in a time-dependent manner in HEC1A cells. In addition, these analogs induced an activation of ERK1/2 and p38 MAPK in a time-dependent manner as downstream pathways of FAK. It appears that GnRH-II has much greater effect on FAK, ERK1/2 and p38 MAPK than GnRH-I in these cells. Further, the growth inhibition of HEC1A cells by GnRH-I or GnRH-II may be involved in the activation of integrin-FAK, ERK1/2 and p38 MAPK pathways. Thus, these results suggest that GnRH may have an effect on the inhibition of cell growth of endometrial cancer cells through a direct pathway. This knowledge could contribute to a better understanding of the mechanisms implicated in the action of GnRH and its biomedical application for the treatment against endometrial cancer. Although this study showed an inhibitory effect of GnRH on endometrial cancer cell growth, a further study is essential to reveal the ultimate cellular change and effect of GnRH on endometrial cancer cells.

## Competing interests

The authors declare that they have no competing interests.

## Authors' contributions

DWP carried out the overall experiments to complete this study and drafted the manuscript. KCC performed experiments, in part, and drafted and finalized the manuscript. CDM participated in the design of the study and performed the statistical analysis. PCKL conceived of the study, and participated in its design and coordination and helped to draft the manuscript. All authors read and approved the final manuscript.
